# Some New Methodological and Conceptual Aspects of the “Acid Growth Theory” for the Auxin Action in Maize (*Zea mays* L.) Coleoptile Segments: Do Acid- and Auxin-Induced Rapid Growth Differ in Their Mechanisms?

**DOI:** 10.3390/ijms22052317

**Published:** 2021-02-26

**Authors:** Małgorzata Polak, Waldemar Karcz

**Affiliations:** Institute of Biology, Biotechnology and Environmental Protection, Faculty of Natural Sciences, University of Silesia in Katowice, Jagiellońska 28, PL-40032 Katowice, Poland; malgorzata.m.polak@us.edu.pl

**Keywords:** auxin, coleoptile segments, growth, membrane potential, proton extrusion

## Abstract

Two arguments against the “acid growth theory” of auxin-induced growth were re-examined. First, the lack of a correlation between the IAA-induced growth and medium acidification, which is mainly due to the cuticle, which is a barrier for proton diffusion. Second, acid- and the IAA-induced growth are additive processes, which means that acid and the IAA act via different mechanisms. Here, growth, medium pH, and membrane potential (in some experiments) were simultaneously measured using non-abraded and non-peeled segments but with the incubation medium having access to their lumen. Using such an approach significantly enhances both the IAA-induced growth and proton extrusion (similar to that of abraded segments). Staining the cuticle on the outer and inner epidermis of the coleoptile segments showed that the cuticle architecture differs on both sides of the segments. The dose-response curves for the IAA-induced growth and proton extrusion were bell-shaped with the maximum at 10^−4^ M over 10 h. The kinetics of the IAA-induced hyperpolarisation was similar to that of the rapid phase of the IAA-induced growth. It is also proposed that the K^+^/H^+^ co-transporters are involved in acid-induced growth and that the combined effect of the K^+^ channels and K^+^/ H^+^ co-transporters is responsible for the IAA-induced growth. These findings support the “acid growth theory” of auxin action.

## 1. Introduction

Historically, the “acid growth theory” of auxin-induced growth goes back to the pioneering studies of Robert Cleland [1,2] and Achim Hager [3,4]. Briefly, in agreement with the theory, auxin induces growth via the acidification of the cell walls, which leads to wall loosening and growth. Since the “acid growth theory” of auxin-induced growth was proposed, much evidence both for [5,6,7,8] and against it [9,10,11,12,13,14,15], as well as a large amount of new information on the mechanisms of auxin action on cell growth, has been reported [16,17,18,19,20]. The criticism of the “acid growth theory” of auxin-induced growth primarily concerns the lack of quantitative relationships between the IAA-induced growth and proton extrusion, which was predominantly connected with the methods that were used to circumvent the cuticular barrier for proton diffusion. Another argument against this theory was that the acid- and IAA-induced growth are additive processes, which means that acid and the IAA act via different mechanisms [10,12]. The latter problems are not resolved to date.

At present, it is well established that the auxin induces rapid cell elongation in coleoptile segments within minutes after auxin has been added (reviewed in [4,8,12,21]). This rapid effect is believed to be the result of the activation of the PM H^+^-ATPase (proton pump), which acidifies the apoplast and causes the activation of the enzymes that are involved in cell wall loosening (reviewed in [4]). The activation of the PM H^+^-ATPase by auxin causes the hyperpolarisation of the membrane potential [22,23,24,25,26] and, as was shown in patch-clamp experiments, the activation of the voltage-dependent K^+^ inward channels [27,28,29,30]. The uptake of potassium through the K^+^ channels contributes to the water uptake that is necessary to sustain cell expansion. It has also been shown that apart from the posttranslational, auxin-dependent up-regulation of both the K^+^ uptake channels and the PM H^+^-ATPases, auxin also regulates their expression [28,31]. Quite recently, it was also shown that the SV (slow vacuolar) and FV (fast vacuolar) channels, which represent the conductance of the major cations across the tonoplast, are involved in the IAA-induced volume changes of the vacuoles [32,33]. 

Although much is known about the effect of the IAA on the growth of coleoptiles, many important questions remain unanswered. Here, we focused on two questions. First, whether abrasion and peeling, the methods that are used to circumvent the cuticular barrier for H^+^ ions diffusion are the only methods that can be used to enhance the diffusion of the protons from the apoplast. Second, what are the mechanisms of the acid- and IAA-induced rapid growth? In this study, using non-abraded and non-peeled coleoptile segments, but with the incubation medium having access to their lumen, some methodological and conceptual problems of the “acid growth theory” of auxin-induced growth were examined. The results presented here clearly show that the access of the incubation medium to the lumen of the coleoptile segments significantly enhanced both the IAA-induced growth and proton extrusion (similar to that of the abraded coleoptile segments) and this finding may solve the problems that are connected with the abrasion and peeling procedures. However, as for the additivity of acid- and auxin-induced growth, it may be suggested that the K^+^/H^+^ co-transporters are responsible for acid-induced rapid growth, while the combined effect of the K^+^ channels and the K^+^/H^+^ co-transporters is involved in auxin-induced growth. 

## 2. Results

### 2.1. Methodological Aspects of the Experiments with Maize Coleoptile Segments

**Figure 1:**
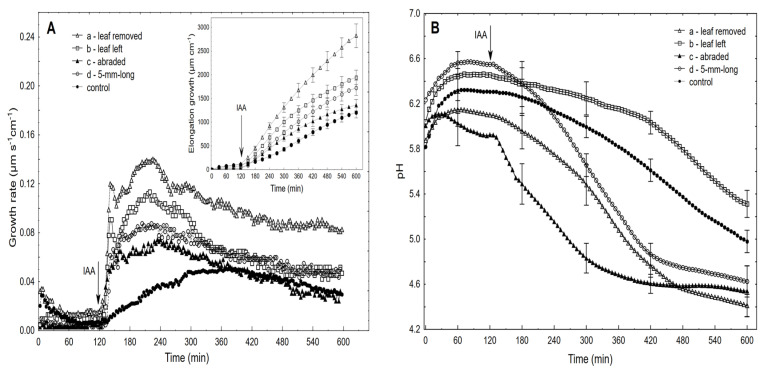
Effect of the IAA (10^−4^ M) on the growth rate (µm s^−1^ cm^−1^) and medium pH of the maize coleoptile segments. The IAA-induced growth (**A**) and medium pH (**B**) of the coleoptile segments: with (a) or without (b) the first leaf removed, with a partially abraded cuticle (c) and segments cut to half of their length (5-mm-long segments) (d). The control represents the growth rate of the coleoptile segments in the auxin-free medium (endogenous growth). The coleoptile segments were first preincubated (over 2 h) in an auxin-free medium to which the IAA at a final concentration of 10^−4^ M was added (arrow). The inset shows the total IAA-induced elongation growth, which was calculated as the sum of the extension from 3 min interval measurements over 10 h. All of the curves are the means of at least eight independent experiments. Bars indicate ± SE.

We used 10-mm-long coleoptile segments, which had been cut off from the subapical region of the coleoptiles, from which the first leaf had been removed. The growth experiments with the coleoptile segments were carried out in an apparatus that enabled the elongation growth, medium pH and membrane potential of coleoptile cells from the same tissue sample to be simultaneously measured (Figure 9, Section 4). In our measuring system, the incubation medium flowed through the lumen of the coleoptile cylinders. This feature enabled the experimental solutions to be in direct contact with the interior of the segments, which significantly enhances both the elongation growth of the coleoptile segments and proton extrusion, as was previously shown with FC in short-term experiments [34]. Here, we extended our previous findings in experiments that were performed using a new measuring system (Figure 9) in which long-term (10 h) experiments with the IAA, which was added at the optimal concentrations of 10^−4^ M (see the next section), were carried out. Figure 1 presents (for comparison) the results of the experiments in which the effect of auxin on the growth and medium pH of the segments: with or without the first leaf having been removed (segments with or without access of the incubation medium to their lumen, respectively), with a partially abraded cuticle (abrasion creates scattered holes in the cuticle) and segments that were cut to half of their length (5-mm-long segments, two-fold increase of cuts) was studied. 

The latter two variants of the experiments were performed in order to show that the solution that was used here (the incubation medium had access to the lumen of the coleoptile segments) might be successful substitutes for the methods that are used to enhance proton diffusion from apoplast of the cells. As can be seen from Figure 1A,B, the IAA-induced growth and medium acidification were significantly greater in the segments with the first leaf removed compared to the segments with the first leaf left. For example, in the presence of the IAA, the total elongation growth of the maize coleoptile segments with the first leaf removed (Figure 1A, inset) was about 30% greater, and the medium pH was about ca. 1.0 unit lower (from pH 5.4 to pH 4.4; Figure 1B) compared to the segments with the first leaf left, respectively. The latter means that the H^+^ concentration *per* coleoptile segment at 10 h was ca. ten-fold greater for the segments with the first leaf removed than for segments with the leaf left. The abrasion of the coleoptile segments with SiC powder (1200 mesh), which was done in accordance with the protocol that was described by Hager et al. [35], caused about ca. 50% decrease in the total IAA-induced elongation growth and strong acceleration of the IAA-induced proton extrusion, especially within the first three hours. This finding is in good agreement with the data that was presented by Hager et al. [35]. Interestingly, in the abraded coleoptile segments, the acidification of the incubation medium began before the IAA was added, and after 10 h, it was similar to that of the non-abraded segments (Figure 1B). To increase the IAA-induced proton extrusion, the segments were also cut to half of their length (5-mm-long segments) in order to double the number of cuts across which the diffusion of protons is facilitated. In this case, the IAA-induced proton extrusion significantly increased after the IAA was added and at 10 h, it reached a level that was similar to that of the abraded and non-abraded segments from which the first leaf had been removed. However, the IAA-induced total elongation growth of the 5-mm-long coleoptile segments was two-fold lower compared to the 10-mm-long segments (for comparison, see [36]) and was similar to that of the abraded segments. It should also be added that the alkalisation of the incubation medium by the 5-mm-long segments, which was observed before the IAA was added, was very high, which might suggest that the additional cuts caused strong stress. 

**Figure 2:**
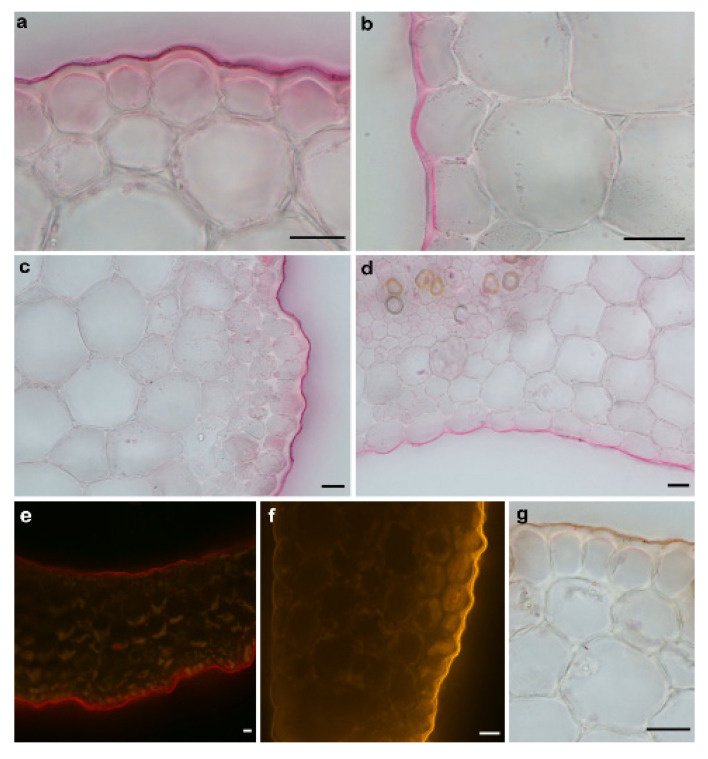
Light microscopy photographs of the cross-sections of the maize coleoptile segments (*Zea mays* L.) that had been stained with the lipophilic dyes in order to visualise the cuticle. (**a**,**c**) and (**b**,**d**) The cuticle on the outer and inner epidermis of the coleoptile segments that had been stained with Sudan Red 7B under a bright field microscope, respectively. (**e**,**f**) After Sudan IV and Nile Red staining under an epifluorescence microscope. The cuticle was observed as a continuous bright red layer or orange-gold, respectively, on both the outer and inner epidermal surfaces. (**g**) Sudan IV rapid routine staining to identify the cuticle on the outer epidermal surface. Scale bars: (**a**–**g**) 20 µm.

Taking into account that the access of the incubation medium to the lumen of the segments significantly enhanced their elongation growth and proton extrusion, we began to explore this phenomenon because we suspected that it was connected with the different architecture of the cuticle that covers the outer and inner epidermis of the maize coleoptiles. To prove this, histochemical analysis of the cuticular barrier on both sides of the coleoptile segments was performed (Figure 2).

As can be seen in Figure 2, the cuticle proper and the cuticular layer, which are the components of the cuticle (for an explanation of the terminology, see [37,38]), were clearly visible outside of the cell walls of the outer epidermis of the coleoptile segments and were present as a conspicuous bright layer. However, both components of the cuticle on the inner epidermal cells were continuations of the underlying cells of the epidermis, and their boundaries could not be delineated. This may suggest that the cuticle on the inner epidermal cells does not form a separate layer but might be embedded within the cell walls. Moreover, as can be seen in Figure 2, another important difference between the cuticle on the outer and inner epidermal cells is that the latter is much thinner and also differs in its architecture. 

### 2.2. The Dose-Response Curves for the IAA-Induced Elongation Growth of Coleoptile Segments and Medium pH Measured Simultaneously with the Growth 

**Figure 3:**
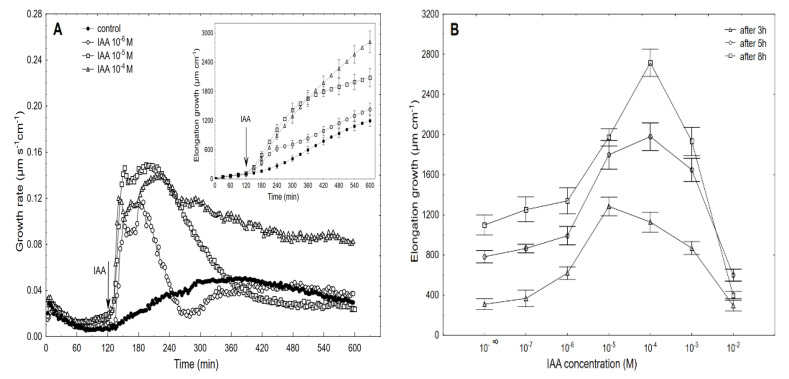
Growth rate (µm s^−1^ cm^−1^) of the maize coleoptile segments that had been incubated in the presence of 10^−6^, 10^−5^ and 10^−4^ M of the IAA (**A**) and the dose-response curves for the IAA (10^−7^–10^−2^ M)-induced total elongation growth of the coleoptile segments as a function of time (**B**). The coleoptile segments were first preincubated (over 2 h) in an auxin-free medium to which the IAA was added (arrow). The inset shows the total elongation growth (µm cm^−1^), which was calculated as the sum of the extensions from 3 min interval measurements over 10 h. All of the curves are the means of at least six independent experiments. Bars indicate ± SE.

High-resolution measurements of the growth were performed using an angular position transducer, which resulted in a precise record of the growth kinetics of the maize coleoptile segments. Figure 3 shows the growth-promoting activity of 10^−6^, 10^−5^ and 10^−4^ M of the IAA (shown as an example) and the dose-response curves for the IAA (10^−7^–10^−2^ M)-induced total elongation growth of the coleoptile segments as a function of time.

**Figure 4:**
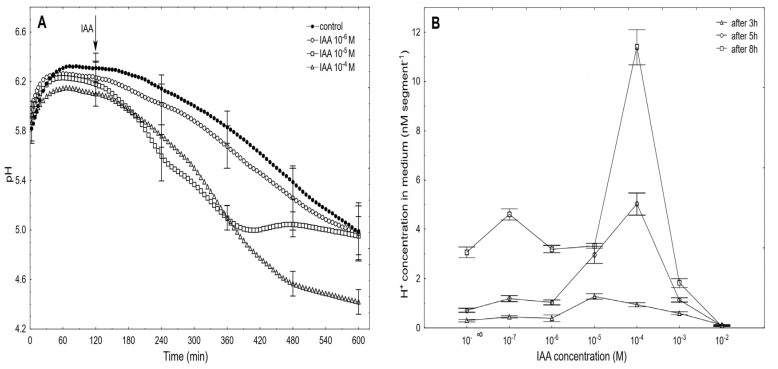
Kinetics of the medium pH changes of the coleoptile segments that had been incubated in the presence of 10^–6^, 10^–5^ and 10^–4^ M of the IAA (**A**) and dose-response curves for the IAA (10^–7^–10^–2^ M)-induced medium pH changes (expressed as changes in the H^+^ concentration *per* coleoptile segment) of the coleoptile segments as a function of time (**B**). The coleoptile segments were first preincubated (over 2 h) in an auxin-free medium to which the IAA was added (arrow). The pH values are the means of at least six independent experiments that were performed simultaneously with growth (shown in Figure 3) using the same tissue samples. Bars indicate ± SE.

When auxin was added to the control medium, it induced strong growth, the kinetics of which could be separated into two phases. The first phase (very rapid) was followed by a long-lasting one that began about 30 min after the IAA was added (Figure 3A). For example, the maximal growth rate of about 0.15 µm s^−1^ cm^−1^ was observed at 10^−5^ M of the IAA for both growth rate phases, while at 10^−6^ and 10^−4^ M of the IAA, the growth rate of these phases differed. Based on the growth rate responses that are shown in Figure 3A (to ensure the clarity of the figure, only four of the seven curves are shown), the total elongation growth over 10 h was calculated (Figure 3A, inset). Taking into account the results of all of the growth experiments that were performed in a wide range of the IAA concentrations (10^–7^–10^–2^ M), the dose-response curves for the IAA-induced elongation growth (elongation measured 3, 5 and 8 h after addition of IAA) were constructed (Figure 3B). The data in Figure 3B indicate that when the maize coleoptile segments were incubated in the presence of the various IAA concentrations, there were bell-shaped dose-response curves, regardless of the duration of the incubation of the segments in the presence of the IAA. However, as can be seen in Figure 3B, the IAA at 10^–5^ M was optimal in the short-term experiments (3 h), while the IAA at 10^–4^ M was optimal in the longer ones (5 and 8 h). For example, over 8 h, the IAA (10^–4^ M)-induced elongation growth of the coleoptile segments was about 40% higher than for the IAA at 10^–5^ M. The data that was obtained for medium pH (Figure 4), which was measured simultaneously with growth, indicated that the coleoptile segments that had been incubated in the control medium characteristically changed its pH.

Generally, within the first two to three hours, there was an increase of pH to 6.0–6.4, which was followed by a slow decrease to a pH of approximately 5.0 after 10 h. After the IAA was added to a medium, its effect on the medium pH depended on the IAA concentration and time. At the highest concentration (10^–2^ M), the IAA caused medium alkalisation (data are shown in Figure 4B), while at the rest of the concentrations, there was acidification of the medium. Figure 4A shows that the kinetics of the IAA (10^–6^ M)-induced medium pH changed in a way that was similar to the change that was observed in the control medium. However, when the IAA at 10^–5^ M was added to the control medium at 120 min, there was an additional decrease in pH to ca. 5.0 within the next 220 min (up to 340 min), while after this time, there was a recovery of medium pH to a value of the control medium. A similar recovery of the medium pH was observed by others [10,12,39] as well as by us [25]. However, as was shown here, this recovery of the medium pH was due to the suboptimal concentration of the IAA that was used, because when the IAA was added at a final concentration of 10^–4^ M IAA, there was a decrease in the medium pH to approximately 4.5 after 10 h. At 10^–4^ M of the IAA, the kinetics of the medium pH changes within the first 220 min (up to 360 min) was similar to the one that was observed at 10^–5^ M of the IAA, which suggests that within the first three to four hours, the IAA at 10^–5^ M is optimal. However, in longer experiments, a higher concentration of IAA should be used, especially considering the volume of the medium per coleoptile segment. Based on the results of all of the pH experiments, the dose-response curves for the IAA (10^–7^–10^–2^ M)-induced medium pH changes (expressed as H+ concentration per coleoptile segment) as a function of time were constructed (Figure 4B). As can be seen in Figure 4B, the IAA at 10^–4^ M was optimal for the IAA-induced proton extrusion per coleoptile segment in long-term experiments (5 and 8 h), while the IAA at 10^–5^ M was enough in the short-term experiment (3 h). As can be seen from Figure 3 and Figure 4, the IAA at 10^–4^ M was optimal for either the total IAA-induced elongation growth or the IAA-induced proton extrusion in long-term experiments (5 and 8 h). The correlations between the IAA-induced growth and medium acidification that was calculated at the optimal IAA concentration (10^–4^ M, over 8 h) confirm the validity (Pearson correlation coefficient equal 0.91) of the “acid growth theory” of auxin action. 

### 2.3. Effect of Auxin on the Membrane Potential (E_m_) of the Parenchymal Cells Simultaneously Measured with the Growth and Medium pH of the Coleoptile Segments.

**Figure 5:**
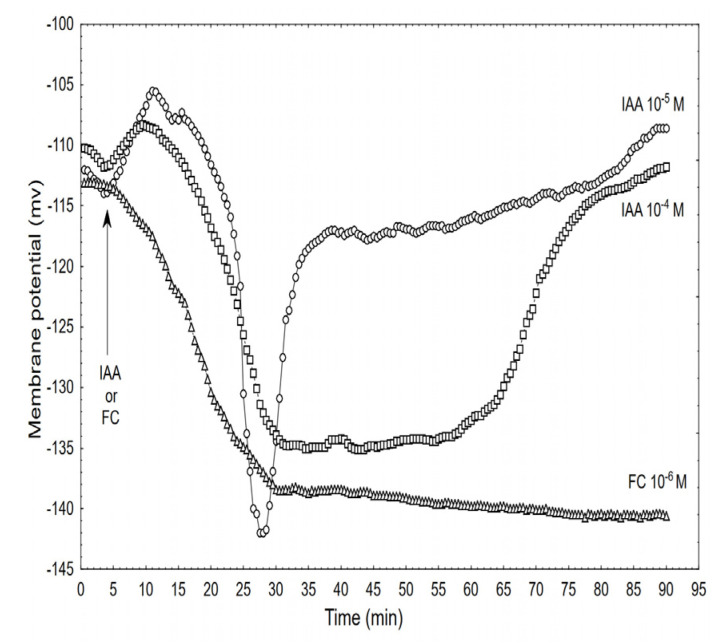
Effect of the IAA (10^–5^ and 10^–4^ M) and FC (10^–6^ M) on the membrane potential (E_m_) of the parenchymal coleoptile cells simultaneously measured with the growth and medium pH. At 5 min (arrow), the IAA or FC was added to the growth medium. The typical results that were selected from at least seven experiments is presented. The adequate mean values are indicated in the text.

The mean (E_m_) of the parenchymal coleoptile cells that was simultaneously measured with the growth and medium pH in the control medium (Figure 5) was −113.3 ± 4.1 mV (mean ± SE, *n* = 12).

Adding the IAA to the control medium at 10^–5^ M caused changes in the membrane potential of the parenchymal cells that included: initial, transient depolarisation by ca. 9.0 ± 1.8 mV (mean ± SE, *n* = 7), which was followed by a rapid (within ca. 8 min) hyperpolarisation of the E_m_ during which the membrane potential was 27 ± 2.1 mV more negative than the original value (−113.3 ± 4.1 mV) after which the hyperpolarisation of the membrane rapidly decreased (within 5-6 min) to about −120 mV. Within the next 30 min, the membrane potential returned to the original value. However, when the coleoptile segments were treated with the IAA at 10^−4^ M, the character of the auxin-induced membrane potential changes was generally similar to the one that was observed at 10^−5^ M IAA, although it differed in the details: at 10^−4^ M of the IAA, the rapid hyperpolarisation was somewhat slower and by ca. 7 mV lower than at 10^−5^ M of the IAA, however, its duration increased by about 25 min. For comparison, the effect of the FC at 10^−6^ M is also shown. When the FC was added to the medium, it caused a rapid (with kinetics similar to those for 10^−4^ M of the IAA) hyperpolarisation of the E_m_, which at 30 min was about −139 mV and did not change significantly within the next 45 min. 

### 2.4. The Effects of Acid and Acid Combined with Auxin on the Growth and Membrane Potential of Coleoptile

**Figure 6:**
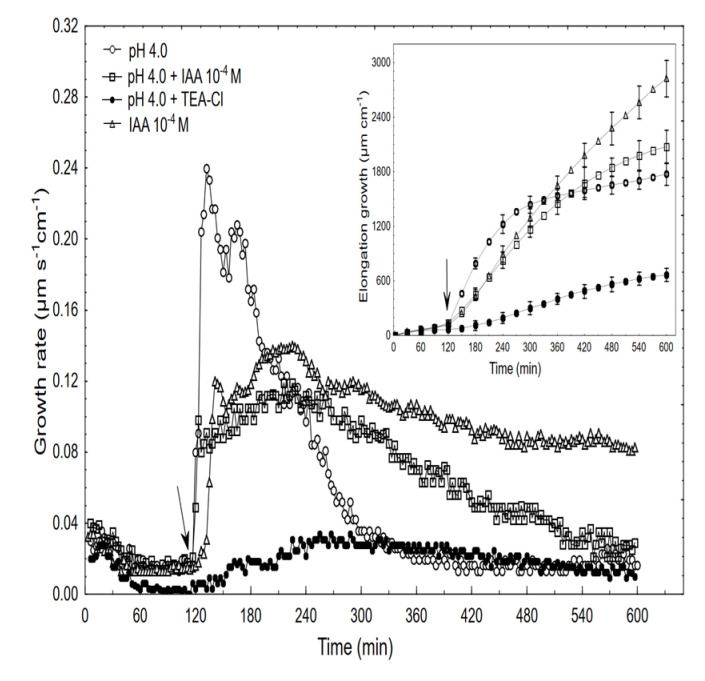
Effect of the acid buffer (pH 4) and its combination with the IAA or TEA-Cl on the growth rate (µm s^−1^ cm^−1^) and elongation (µm cm^−1^) of the maize coleoptile segments. The coleoptile segments were first preincubated (over 2 h) in the control, after which this medium was changed for a new one containing the acid buffer or its combination with the IAA (10^−4^ M) or TEA-Cl (30 mM). The inset on the right side shows the total elongation over 10 h. The typical results were selected from six experiments. The adequate mean values are indicated in the text.

As is presented in Figure 6, an acid buffer (pH 4, citrate buffer) within the first 12 min caused the rapid and strong growth of the coleoptile segments (with a maximal growth rate of about 0.24 µm s^−1^ cm^−1^) after which the growth rate decreased significantly (exponentially) and reached the minimum at ca. 300 min.

**Figure 7:**
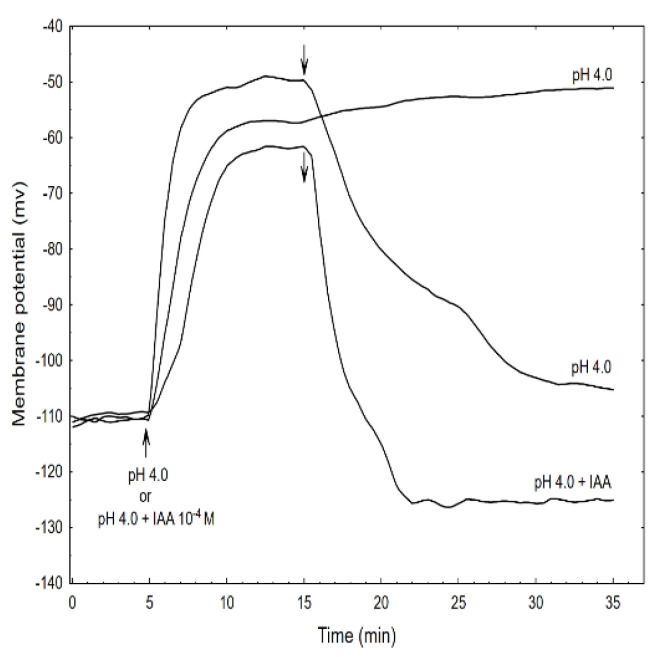
Effect of the acid buffer (pH 4) and its combination with the IAA on the membrane potential (E_m_) of the parenchymal coleoptile cells. At time 5 min (arrow), the control medium was changed for a new one with the same salt composition, but that also contained the acid buffer (pH) or its combination with the IAA (10^−4^ M). An arrow pointing down indicates the exchange of the medium for the control medium. The representative curves for each variant are shown. The adequate mean values are indicated in the text.

When comparing the acid- and auxin (10^−4^ M)-induced growth rate kinetics, it should be pointed out that in the first case, growth began without a lag phase and its amplitude was about two-fold higher than for auxin. However, when the combined effect of the acid and the IAA was studied, the growth rate kinetics consisted of two phases: the first was very rapid (without lag phase) but had a significantly lower amplitude compared to the acid-induced growth, after which the second phase, which is characteristic of the auxin-induced growth, was observed. The data in Figure 6 also shows that when TEA-Cl (K^+^ uptake blocker) was applied together with the acid buffer, it eliminated the acid growth of the coleoptile segments. Similarly, as was previously shown by us and others, the TEA-Cl at 30 mM also eliminated the IAA-induced growth and proton extrusion [40,41,42], which simultaneously caused the membrane hyperpolarisation [26,43]. The inset in Figure 6 clearly shows that neither acid- nor auxin-induced growth are additive processes, which suggests that both have the same mechanism or that the mechanisms of both overlaps. When comparing the acid- and IAA-induced growth, it seems correct to compare the total elongation rather than only the growth rate dynamic (see, [10]). Replacing the control medium with the acid buffer caused the rapid depolarisation of E_m_ by 56.3 ± 4.8 mV (mean ± SE, n = 11) and stabilised its value at a new level (Figure 7).

This effect was reversible because replacing the acid buffer with the control medium repolarised the membrane potential (within 15 min) to approximately the original value. However, the addition of the acid buffer combined with the IAA caused the depolarisation of E_m_ by 45.2 ± 5.2 mV (mean ± SE, *n* = 5) which value was significantly different (*t*-test) from depolarisation induced by acid alone (54.7 ± 7.1 mV, mean ± SE, *n* = 5). In turn, when the acid combined with the IAA was replaced with the control medium, the repolarisation of E_m_ was 17.6 ± 2.2 mV (mean ± SE, *n* = 5) less than its original value. It is well established that the negative electrical potential across the plasma membrane depends on the activity of the H^+^-ATPase (proton pump), which exports H^+^ from the cytoplasm to an external medium (reviewed in [44]). This pump energizes the K^+^-uptake by both the K^+^ channels and the K^+^/H^+^ co-transporters [45,46]. Auxin causes the hyperpolarisation of the plasma membrane by activating the proton pump; however, as was shown here and by others [47,48], the acid buffer causes the depolarisation of the membrane, which is reversible in an alkalised medium. However, when the acid buffer combined with the IAA was replaced with the control medium, the repolarisation of the membrane potential to a more negative value (by 17.6 mV) than the original potential was observed. 

## 3. Discussion

In this study we selected the elongation growth rate of the maize coleoptile segments as the main parameter in order to determine some aspects of the auxin action on plant cell growth. In addition to growth, medium pH and membrane potential were also measured. The relationships between these three parameters are fundamental for the “acid growth theory” of auxin-induced growth (reviewed in [4]). It should also be added that the coleoptile represents a classic model system for studies on the elongation growth of plant cells and that most of the crucial evidence on the mechanisms of auxin action was obtained from coleoptile segments (reviewed in [4,8,10,49]). As was also stated in these articles, the contradictions that exist in the literature regarding the causal relationships between auxin-induced apoplast pH-drops and auxin-mediated growth, which raise the question about the correctness of the “acid growth theory” of auxin action, concern the methods that are used to circumvent the cuticular barrier for the H^+^ ions, the protocols for preincubating the segments before the growth effectors are added, the volume of the medium per coleoptile segments, the concentrations of the growth effectors and potassium in the incubation medium and many other details. The experiments presented here clearly show that the access of the incubation medium to the lumen of the coleoptile segments significantly enhanced both the IAA-induced growth and proton extrusion (similar to that of the abraded coleoptile segments), and this finding may solve the problems that are connected with the abrasion and peeling procedures (Figure 1). In addition, this finding also creates proper conditions to determine the correlation between growth and medium acidification in contrast to the abraded or peeled coleoptile segments for which the IAA-induced growth was significantly decreased, while the proton extrusion was significantly increased. The correlations between both parameters, which were calculated here at the optimal IAA concentration (10^−4^ M, over 8 h), confirm the validity (Pearson correlation coefficient equal 0.91) of the “acid growth theory” of auxin action.

Staining the cuticle on the outer and inner epidermis of the coleoptile segments showed that the cuticle architecture differs on both sides of the segments (Figure 2), which probably explains the massive IAA-induced proton diffusion of protons from the lumen of the coleoptile segments. In addition, it might be suggested that because the lumen of the coleoptiles forms a small closed space (which also contains the first leaf), the requirements for the cuticle to act as a barrier to the penetration of water and ions [50,51,52] are less inside of than outside of the maize coleoptiles.

The dose-response curves for the IAA-induced growth and proton extrusion are bell-shaped with the optimum at 10^−4^ M, over 10 h (Figure 3 and Figure 4). Bell-shaped dose-response curves for the IAA-induced growth of the coleoptile segments were also reported by a number of researchers [5,53,54,55,56]. Because of the different experimental conditions (e.g., the number of coleoptile segments *per* medium volume, the composition of the incubation medium and its initial pH, the concentrations of the growth effectors, etc.), a direct quantitative comparison of our data with the results that have been presented by other authors is difficult. In literature little attention has so far been paid to detailed studies of the medium pH changes (measured simultaneously with growth) that are caused by a wide range of IAA concentrations. Here, acidification of the incubation medium was found at all of the concentrations, except for 10^−2^ M of the IAA that was used and the bell-shaped dose-response curves for the IAA-induced proton extrusion (Figure 4) resembled those for the elongation growth. 

To the best of our knowledge, the growth, medium pH and membrane potential have never been measured simultaneously. Generally, the characteristic IAA-induced changes in the membrane potential of the coleoptile cells that were observed here (the initial, transient depolarisation followed by the hyperpolarisation of the membrane) are in good agreement with other authors [22,23,24,25,42,57,58]. However, taking into account our experimental conditions (membrane potential was measured simultaneously with the growth and medium pH), it should be pointed out that these characteristic changes differed in their kinetics compared to those in the articles cited above. First of all, the IAA-induced membrane hyperpolarisation was very rapid and transient with kinetics similar to those of the first phase of the IAA-induced growth (Figure 5). At present, there is no doubt that the hyperpolarisation plasma membrane is a result of the stimulated proton extrusion through H^+^-ATPase [27,59]. For the IAA-induced initial depolarisation, however, several mechanisms including the activation of the anion channels [24], the activation of the nH^+^/IAA-symporter [23], unspecific weak acid effects [60] and auxin transport [61] have been proposed. Interestingly, in contrast to the IAA, chlorinated auxin (4-Cl-IAA) caused the immediate hyperpolarisation of the membrane potential that was two-fold greater than for the IAA [25].

**Figure 8:**
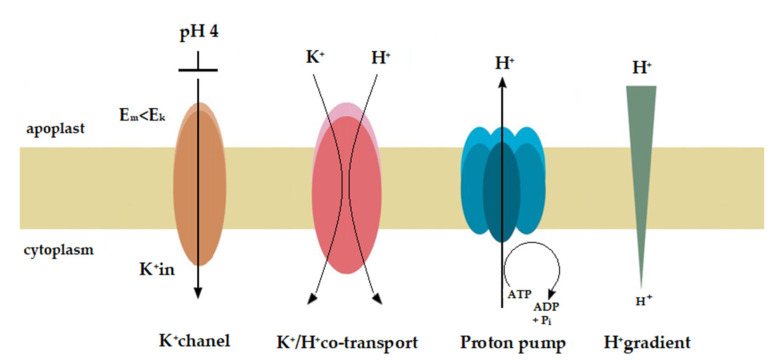
Hypothetical model for the molecular mechanisms of acid- and auxin-induced rapid growth. This model assumes that the K^+^/H^+^ co-transporters are responsible for the acid-induced rapid growth, while both the K^+^ channels and K^+^/H^+^ co-transporters are involved in the auxin-induced growth. On the basis of the available and our own results, we speculate that at high H^+^ concentration in the medium (pH 4) the proton pump current amplitude decreases and the membrane potential (E_m_) shifts to less negative values (here from −110 to ca. −50 mV, Figure 7). At a K^+^ concentration in the cytosol of 100 mM and an extracellular K^+^ concentration of 1 mM (in our experiments) the equilibrium (Nernst) potential for K^+^ (E_K_) is equal −118 mV, suggesting that at E_m_ more positive than E_K_ (here E_m_ about −50 mV at pH 4) the K^+^ rectifying inward channels are blocked. Taking into account that an acid buffer (pH 4) caused the rapid and strong growth of the coleoptile segments, it may be suggested, that the K^+^/H^+^ co-transporters are responsible for the acid-induced growth. However, when auxin was added to the incubation medium, it induced (after initial depolarization) a rapid (transient) hyperpolarisation of the E_m_ (Figure 5) during which the membrane potential was by ca. 20 mV more negative than E_K_. This means that in the presence of IAA the inward rectifying K^+^ channels are open and can mediate K^+^ uptake into the cell. Because the kinetics of the IAA-induced hyperpolarisation is similar to that of the first (very rapid) phase of the IAA-induced growth it may be suggested that the inward rectifying K^+^ channels are involved in the phase. The onset of the second phase of IAA-induced growth rate (Figure 3) correlates in time with rapid depolarisation of the E_m_ observed here after the maximum of the IAA-induced membrane hyperpolarisation was achieved. This finding (transient membrane hyperpolarisation) may indicate that the K^+^/H^+^ co-transporters are responsible for the second phase of the IAA-induced growth.

Taking into account both the growth and electrophysiological experiments, the question may be raised of whether auxin and acid induce the same mechanisms to stimulate elongation growth. In our opinion, the answer to this question was partly resolved in a recent paper by Dreyer and Michard [62], although it deals with a completely different problem. In in silico experiments, the authors simulated the dependency of the two K^+^-uptake modules, the “pump & K^+^ channel module” and the “pump & K^+^/H^+^ co-transporter module” on the external K^+^ concentration and external pH. What followed from the in silico experiments, and is useful in answering the question we posed, is that the activity of the proton pump inevitably depended on the membrane potential and proton concentration on both sides of the plasma membrane. To enable the K^+^-uptake by a K^+^ channel, the membrane potential had to be more negative than the equilibrium potential for potassium; however, the higher the concentration of protons in the external medium, the more difficult it was for the proton pump to establish a sufficiently negative membrane potential (Figure 2 in [62]). As a consequence of both effects, the channel-based module, in contrast to the co-transporter-based one, has limitations for the K^+^-uptake. At very low K^+^ external concentrations and/or high H^+^ external concentrations, the energy from ATP-hydrolysis is not sufficient enough to energize the combined K^+^-uptake/H^+^-release via the pump & K^+^ channel module (Figures 3 and 4 in [62]). Under our experimental conditions, this may mean that the K^+^/H^+^ co-transporters are possibly responsible for the acid-induced rapid growth, while both the K^+^ channels and K^+^/H^+^ co-transporters are involved in the auxin-induced growth (Figure 8). 

It is possible that the discrimination between K^+^ channels and K^+^/H^+^ co-transporters in the auxin-induced growth depends on the pH of the apoplast and the external K^+^ concentration, which, in turn, have an impact on the membrane potential. The hypothesis proposed above can also explain why the IAA-induced proton extrusion in media containing Na^+^ is inhibited compared with K^+^, although the IAA-induced growth is not significantly changed when K^+^ is replaced by Na^+^. This finding was used by Kutschera and Schopfer [10] as an argument against the “acid growth theory” of auxin action. In our opinion, when K^+^ is replaced in the medium by Na^+^, the ionic mechanism of IAA action is dominated by Na^+^/H^+^ co-transporter that mediates Na^+^ uptake by symport with H^+^. This scenario may explain why Na^+^ strongly inhibited medium acidification and had no effect on the growth of plant cells. Support for our hypothesis may be drawn from experiments performed with the root cells of *Arabidopsis* lacking AKT1 inward-rectifying K^+^ channels ([63] and literature cited therein). These authors showed that the inward-rectifying K^+^ channels mediate the K^+^ uptake across the plasma membrane of the root cells in parallel with one or more genetically distinct K^+^ transporters. 

It should be also added that the subapical segments of hypocotyls/epicotyls of dicot seedlings are also used following essentially the same protocol as for coleoptile segments. The auxin-induced growth of cut stem segments follows a biphasic curve. The first phase in the growth rate after a lag period is mimicked by weak acids or buffers at an acidic pH, but the second phase is typical of auxin action and can be sustained for many hours (reviewed in [64]). The above may also suggest that the hypothetical model proposed here for the molecular mechanisms of acid- and auxin-induced rapid growth can also be applied to dicots. In the future experiments we would like to discriminate between K^+^ channels and K^+^/H^+^ co-transporters in the auxin-induced growth.

## 4. Materials and Methods

### 4.1. Plant Material

Caryopses of maize (*Zea mays* L. cv. Koka) were soaked in tap water, sown on wet lignin in plastic boxes and placed in a growth chamber (Type MIR-553, Sanyo Electric Co., Osaka, Japan) at 27 ± 1.0 °C for four days in the dark. The experiments were performed with 10-mm-long coleoptile segments that had been cut from maize seedlings (length of each coleoptile 2–3 cm). The coleoptile segments from which the first leaves had been removed were excised 3 mm below the tip and collected in an incubation medium comprising (control medium) 1 mM KCl, 0.1 mM NaCl and 0.1 mM CaCl_2_. In all of the experiments, the initial pH of the control medium was adjusted to 5.8–6.0. 

### 4.2. Growth, Medium pH and Membrane Potential Measurements

**Figure 9:**
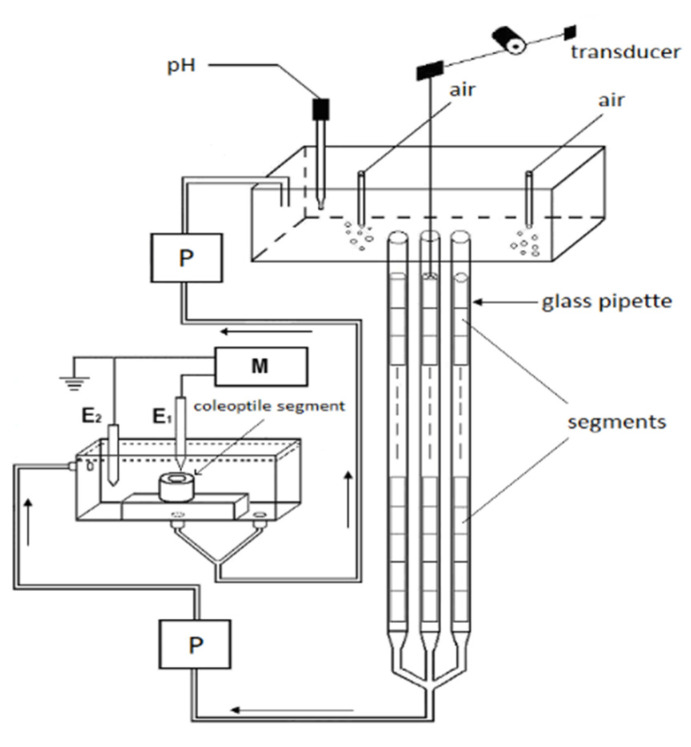
A schematic drawing of the apparatus that was used to simultaneously measure the elongation growth, medium pH and membrane potential of the coleoptile cells. The longitudinal extension of a stack of the segments was recorded in an intensively aerated solution using an angular transducer. The elongation growth, medium pH and membrane potential were measured with multifunctional computer meters: P, peristaltic pump; M, multifunctional computer meter; E_1_ and E_2_, reference and internal electrode, respectively.

The experiments with the coleoptile segments were carried out in an apparatus that enabled their elongation growth, medium pH and membrane potential (in some variants) of the parenchymal cells from the same tissue sample to be simultaneously measured (Figure 9). 

The same apparatus but without an electrophysiological chamber was previously used [42,56,65]. To simultaneously measure growth and medium pH, 60 coleoptile segments were arranged vertically in three narrow glass pipettes (20 segments in each); however, when the electrophysiological chamber was used, each pipette contained 22 segments (in order to increase the volume of the medium). The medium was circulated by a peristaltic pump (1B-05A; Zalimp, Warsaw, Poland). High-resolution measurements of the growth rate were performed using an angular position transducer (TWK-Electronik, Düsseldorf, Germany). The coleoptile segments were incubated in an intensively aerated medium in which the volume of the incubation medium was constant (0.3 mL/segment). The incubation medium also flowed through the lumen of the coleoptile cylinders. This feature enabled the experimental solutions to be in direct contact with the interior of the segments, which significantly enhances both the elongation growth of the coleoptile segments and proton extrusion [34]. The extension growth of a stack of 20 (or 22) segments and the pH of the incubation medium were sampled every 3 min using a multifunctional computer meter (CX-771; Elmetron, Zabrze, Poland). The pH was measured with a pH electrode (OSH 10-10; Metron, Torun, Poland). All of the manipulations, growth and pH measurements were carried out under dim green light at a thermostatically controlled temperature of 25 ± 0.5 °C.

The electrophysiological experiments were performed on the coleoptile segments that were prepared in the same manner as for the growth experiments. A standard electrophysiological technique was used to measure the membrane potential, as was previously described by Karcz and Burdach [25]. Briefly, the membrane potential (E_m_) was measured by recording the voltage between a 3 M KCl-filled glass micropipette that was inserted into the parenchymal cells and a reference electrode in a bathing medium (Figure 8). For the electrophysiological experiments, the segments were placed into a perfusion Plexiglas chamber that was mounted on a vertically placed microscope stage. The microelectrodes were inserted into the cells under a microscope using a micromanipulator (Hugo Sach Electronik, March-Hugstetten, Germany). The micropipettes were made from borosilicate glass capillaries (type 1B150F-3; World Precision Instruments, Sarasota, FL, USA) using a vertical pipette puller (Model PIP 6; HEKA Elektronik, Lambrecht, Germany).

### 4.3. Histochemical Procedures and Staining

For the histochemical procedures, the coleoptile segments (prepared in the same way as for the growth experiments) were placed into a mould and embedded in a 6% solution of agarose at a temperature near the point of solidification (approx. 45 °C). Before pouring the agarose solution in, the coleoptile segments were carefully positioned to be parallel to the longitudinal axis of the mould. The agarose solution was poured into the mould to cover the segments in a 7–10 mm thick gel layer and left to solidify at room temperature. After this step, the segments could be cut or, if necessary, they could be stored at 4 °C for up to a few days prior to being cut. After removing the mould and any excessive agarose around the segments, the samples were cut using a vibratome (Leica VT 1200s). The sections were placed into a drop of water on a slide, and the agarose surrounding the section was manually removed using a preparation needle. The cross-sections for the fluorescence and bright field microscopy were 100 µm thick.

Three stains, namely Sudan IV, Nile Red, and Fat Red (Sudan Red 7B), were used to visualise the cuticle in bright field and fluorescence microscopy. The bright field microscopy and epifluorescence microscopy were performed using a BX41 microscope equipped with an XC50 camera (Olympus, Tokyo, Japan). For the blue light, a filter with a 450–490 nm excitation and a 520 nm emission band was used. The presence of lipid substances was detected in the agarose-embedded sections using Sudan IV (Sigma-Aldrich, St. Louis, MO, USA), Sudan Red 7B (Sigma-Aldrich), and Nile Red (Fluka, Charlotte, NC, USA). 

Sudan IV was used to differentially stain the cuticle using the protocol that was described by Buda et al. [66]. Briefly, a Sudan IV stock solution (0.1% *w*/*v* in isopropyl alcohol) was diluted 3:2 with distilled water, mixed well, allowed to sit at room temperature for 30 min, and filtered through a syringe filter to remove any precipitates. The stain was added to 100 μm sections for 10 min, rinsed first with 50% isopropyl alcohol, and then with distilled water. The slides were mounted in glycerin with a cover slip, sealed with nail polish and viewed immediately using bright field and fluorescence microscopes. Digital images of the sections were analysed using ImageJ software (1.49i) (W.S. Rasband, http://imagej.nih.gov/ij/, accessed on 28 January 2021). After the Sudan IV staining, the cuticle was observed as a continuous bright red layer on both the external and lumen surfaces of the epidermal tissue.

For the Nile Red staining, the protocol that was described by Potocka et al. [67] was used. In accordance with this protocol, the sections were incubated for 10 min in a solution consisting of Nile Red stock (1 mg/mL in acetone) that had been diluted 1:200 in distilled water, quickly rinsed with distilled water and observed using an epifluorescence microscope (excitation filter BP470-490, dichromatic mirror DM500, barrier filter BA520IF, Olympus, Tokyo, Japan). Here, the non-polar or neutral lipids stain orange-gold when examined at specific wavelengths (488 nm).

For the Sudan red 7B staining, the protocol that was described by De Giorgi et al. [68] was used. The samples were stained with Sudan red 7B 0.1% in a 1:1 mix of polyethylene glycol 400 and 90% glycerol for 30 min and then mounted in glycerol. Using Sudan red dye, which specifically stains in pink or red the wax and lipids, we observed a thick extracellular pink-stained layer on the outer side of the coleoptile cross-sections. The cuticular layer inside the coleoptile cross-sections (from the side of the lumen) was much less distinct. Image analysis and quantification were performed using ImageJ software (1.49i) (W.S. Rasband, http://imagej.nih.gov/ij/, accessed on 28 January 2021).

### 4.4. Statistical Analysis

The data were analysed using Statistica software for Windows (STATISTICA Data Analysis Software System, version 13.1 http://www.statsoft.com, accessed on 28 January 2021, Tulsa, OK, USA). The Student’s *t*-test was used to evaluate the significance of the differences between the membrane potential values. The correlation of the elongation growth and proton concentrations was calculated based on Pearson’s correlation. 

## 5. Conclusions

The experiments presented here clearly show that the access of the incubation medium to the lumen of the coleoptile segments significantly enhanced both the IAA-induced growth and proton extrusion (similar to that of the abraded coleoptile segments), and this finding may solve the problems that are connected with the abrasion and peeling procedures. In addition, this finding also creates proper conditions to determine the correlation between growth and medium acidification in contrast to the abraded or peeled coleoptile segments for which the IAA-induced growth was significantly decreased, while the proton extrusion was significantly increased. Staining the cuticle on the outer and inner epidermis of the coleoptile segments showed that the cuticle architecture differs on both sides of the segments, which probably explains the massive diffusion of protons from the lumen of the segments. It was also found that the dose-response curves for the IAA-induced growth and proton extrusion are bell-shaped with the optimum at 10^−4^ M, over 10 h. The correlations between both parameters, which were calculated at the optimal IAA concentration (10^−4^ M), confirm the validity (Pearson correlation coefficient equal 0.91) of the “acid growth theory” of auxin action. The kinetics of the IAA-induced hyperpolarisation of the membrane potential of the parenchymal cells (measured for the first time simultaneously with the growth and medium pH) was similar to that of the rapid phase of IAA-induced growth. A hypothetical model for the molecular mechanisms of acid- and auxin-induced rapid growth was also proposed, which assumes that the K^+^/H^+^ co-transporters are responsible for the acid-induced rapid growth, while both the K^+^ channels and K^+^/H^+^ co-transporters are involved in the auxin-induced growth. 

## Data Availability

The data that support the findings of this study are available from the corresponding author upon reasonable request.
